# Integrating network pharmacology and experimental validation to explore the pharmacological mechanism of Astragaloside IV in alleviating urotensin II-mediated renal tubular epithelial cell injury

**DOI:** 10.1371/journal.pone.0310210

**Published:** 2024-12-20

**Authors:** Wenyuan Liu, Qianwei Liu, Ziyuan Zhang, Yaling Hu, Jingai Fang

**Affiliations:** 1 Department of Nephrology, The First Hospital, Shanxi Medical University, Taiyuan, Shanxi, China; 2 Shanxi Medical University, Taiyuan, Shanxi, China; 3 Department of Nephrology, Beijing Daxing District People’s Hospital, Daxing, Beijing, China; Ondokuz Mayis University Faculty of Medicine: Ondokuz Mayis Universitesi Tip Fakultesi, TÜRKIYE

## Abstract

Renal tubular epithelial cell injury is an important manifestation of chronic kidney disease (CKD). This study aims to explore the mechanism of astragaloside IV (AS-IV) in the treatment of UII-mediated renal tubular epithelial cell injury by integrating network pharmacology and experimental validation. BATMAN, SwissTarget-Prediction and ETCM data bases were used to screen the target proteins of AS-IV. DAVID software was then used to perform GO and KEGG enrichment analysis on these target genes, and STRING and cytoscape were used to construct a protein interaction network. Molecular docking analysis was performed on key genes. The CCK8 assay was applied to detect the cell viability. ELISA, laser confocal, RT-PCR, and Western blot methods were used to detect the expression of cell pathway indicators and inflammatory factors in each group. Network pharmacology analysis found that the cAMP signaling pathway is one of the most important pathways for AS-IV to treat CKD. Molecular docking results showed that the AS-IV can be well embedded in the active pockets of target proteins, such as ALB, VEGFA, AKT1, ROCK1, and DRD2. The cAMP content and expression of GPR-14, PKA, NF-κB, and TGF-β in the UII group and the UII+cAMP agonist group (Forskolin) were all higher than those in the control group (P<0.05). In the UII+SB-611812 group, UII+AS-IV group, UII+losartan group, and UII+cAMP inhibitor (H89) group, the cAMP content and the expressions of GPR-14, PKA, NF-κB and TGF-β were all decreased compared with those in the UII group (P<0.05). In conclusion, AS-IV may improve UII-mediated renal tubular epithelial cell damage by down-regulating the cAMP/PKA signaling pathway.

## 1. Introduction

Chronic kidney disease (CKD) is a condition characterized by a gradual loss of kidney function over a period of months to years. The primary causes include diabetes and hypertension. Patients may experience symptoms such as acidosis, anemia, nerve damage, osteoporosis, and progression of atherosclerosis. Common symptoms include nocturia (increased urination at night), fatigue, nausea, itching, muscle cramps and spasms, loss of appetite, confusion, difficulty breathing, and edema, particularly in the legs. Diagnosis can be confirmed through blood and urine tests. Renal tubular epithelial cells possess high metabolic characteristics, and various injuries such as hypoxia, toxic compounds, proteinuria, and metabolic disorders can lead to damage to these cells. The damage can be categorized as lethal (leading to cell death) and sub-lethal (resulting in injured cells releasing pro-inflammatory or pro-fibrotic mediators or failing to perform their physiological functions), which can further lead to renal fibrosis [1–3].

Our previous research found that in the UUO rat model, the concentration of UII was higher than in the normal group, and subsequent in vitro experiments confirmed that UII can cause pyroptosis of renal tubular epithelial cells [4]. UII as a new vasoactive peptide, can promote the proliferation of various cells in heart and kidney tissues [5–8]. Its receptor is G-protein-coupled-receptor (GPR-14) [6], which is mainly distributed in tissues and organs such as the heart, kidneys, and lungs. The expression of GPR-14 in the kidney is most abundant in the distal convoluted tubule, collecting duct epithelial cells and glomerular capillary endothelial cells [9]. The influence of UII on the kidney may be due to the systemic and local hemodynamic changes it induces, or it may be a direct effect of UII receptor stimulation on glomerular filtration and/or renal tubular secretion and reabsorption [10]. UⅡ can enhance the expression and secretion of TGF-β1 in renal tubular epithelial cells, induce the expression of α-SMA, fibroblast-specific protein 1 (FSP-1), fibronectin and type IV collagen in human renal tubular epithelial cells, and participate in Renal fibrosis process [11–13]. Studies have shown that in addition to regulating vascular tone, UII binding to GPR-14 can also promote cell proliferation and extracellular matrix accumulation in the kidney [7, 12, 14]. Our previous studies found that UII can weaken the L-type calcium current in cardiomyocytes, promote cardiac fibroblast proliferation, and promote cardiomyocyte fibrosis through the cyclic adenosine monophosphate/protein kinase A (cAMP/PKA) signaling pathway [15]. Our further research revealed that UII is also involved in the occurrence and development of renal fibrosis in rats [16]. Our recent preliminary experimental research results show that UII can upregulate the expression of cAMP and NLRP3 in renal tubular epithelial cells. Currently, more and more studies have proven that the cAMP signaling pathway is related to kidney disease [17].

Our previous research found that AS-IV has a certain protective effect on the kidneys of rats with chronic overload stress by reducing the overexpression of UII and collagen I and III in the renal interstitium [16]. AS-IV has various pharmacological effects such as immunomodulation, organ protection, and anti-inflammation [18]. In the chronic pressure overload rat model, astragaloside IV (AS-IV) may delay renal interstitial fibrosis by reducing the overexpression of UII and collagens I and III in the renal interstitium [16]. Studies also have shown [19, 20] that AS-IV can delay the progression of renal fibrosis. The mechanism may be related to the fact that AS-IV participates in the regulation of TLR4/NF-кB, TGF-β/Smad3, and Wnt/β-catenin pathways and can reduce extracellular matrix accumulation and inflammatory cell infiltration in kidney tissue, but the exact mechanism requires further study.

Traditional Chinese medicine or traditional Chinese medicine monomers have the characteristics of complex action targets and action networks. Network pharmacology can obtain complex interaction networks based on target molecules, biological functions, and bioactive compounds. Therefore, network pharmacology research methods are a powerful tool for studying the complex action mechanisms of traditional Chinese medicine, and can elucidate the action mechanism of traditional Chinese medicine formulas at the molecular level from a systematic perspective [21]. This study first intends to construct a "drug ingredient-disease target-signaling pathway" network diagram by exploring the targets of AS-IV in treating CKD. At the same time, this study screened potential target proteins and differentially expressed genes of AS-IV, and further obtained CKD-related target genes by taking the intersection of the two. Then, molecular docking analysis was performed on related genes to elucidate the mechanism of AS-IV in treating CKD and further delaying renal fibrosis from many aspects. This study not only further clarifies the mechanism of action of AS-IV, but also provides a certain theoretical basis for in-depth research and clinical application of AS-IV.

## 2. Material and methods

### 2.1 Materials

Rat renal tubular epithelial cells NRK-52E were purchased from the Cell Bank of the Chinese Academy of Sciences. DMEM high-glucose medium, penicillin-streptomycin solution, sterile PBS solution, trypsin digestion solution (containing EDTA but not phenol red), Certified Fetal Bovine Serum special grade fetal bovine serum, dimethyl sulfoxide DMSO (cell culture grade, CAS: 67-68-5), SB-611812 (urotensin receptor antagonist, CAS: 345892-71-9), astragaloside IV (CAS: 84687-43-4), losartan(CAS: 114798-26-4), H89(CAS: 127243-85-0), and Forskolin (CAS: 66575-29-9) were bought from MCE. UII was obtained from Cloud-Clone Crop. Rabbit anti-NF-κB antibody (ab19870), and rabbit anti-TGF-β antibody (ab215715) were purchased from Abcam. Rabbit anti-PKA antibody, rabbit anti-GPR-14 antibody, and Alexa Fluor® 647 goat anti-rabbit IgG were purchased from Huaan Biotech. 4% paraformaldehyde fixative (CAS: 66455-31-0), RIPA lysis buffer (strong), PMSF were purchased from Beijing Seven Biotech. Horseradish peroxidase-conjugated goat anti-rabbit IgG was purchased from Proteintech. DAPI solution (CAS: 28718-90-3) was purchased from Solarbio. Rat cyclic adenosine monophosphate (cAMP) enzyme-linked immunosorbent assay kit were bought from Jianglaibio. Total RNA extraction kit, and the qPCR amplification kit were bought from Shanghai Promega Biotechnology Company. RNA reverse transcription kit was purchased from Promega. Bovine serum albumin BSA-V(CAS: 9048-46-8) was ordered from Solarbio. Tris-glycine-SDS electrophoresis buffer (10X), Tris-glycine-SDS transfer buffer (10X), TBS-T washing buffer (10X), and SDS-PAGE protein loading buffer (5X), PVDF membrane and filter paper were purchased from Boster Biological Technology.

### 2.2 Prediction and screening of AS-IV target proteins

The BATMAN database [22] was used to obtain potential target proteins existing in Drugbank, KEGG, and TTD databases, and the predicted target proteins were ranked in descending order. In this study, target proteins with scores greater than 5 were selected for further analysis. Set the species of the online tool SwissTargetPrediction [23] to Homo sapiens, enter the simplified molecular formula (SMILES) of AS-IV, and finally select the protein with a Probability score greater than 0 as the target protein. Enter “Astragaloside IV” in the ETCM database [24] to obtain its corresponding target protein. The target proteins predicted using the above three databases were combined as the target of AS-IV.

### 2.3 Experimental data and sources

Four sets of CKD gene data sets numbered GSE15072 [25], GSE62792 [26], GSE66494 [27] and GSE98603 [28] were downloaded from the NCBI GEO [29] database on February 23, 2021.

### 2.4 Screening of significantly differentially expressed genes

For the above four sets of data sets, we first downloaded the preprocessed and standardized probe expression profile files, and downloaded the corresponding probe annotation files to annotate the probes. The probes are then converted into gene symbols, and when different probes map to the same gene symbol, the average value is taken as the expression value of the gene. Finally, the limma package Version 3.34.7 [30] in the R3.6.1 language was used to screen genes (differentially expressed genes, DEGs) that were significantly differentially expressed between CKD and normal groups. In addition, BH is used to correct the pvalue to obtain p.adjust value. The set thresholds are as follows: |log2FC|>0.585 and p.value<0.05.

### 2.5 GO functional analysis and pathway analysis of DEGs

The AS-IV target protein and CKD-related genes obtained through the above steps are intersected to obtain target genes related to AS-IV treatment of CKD. These target genes were then analyzed using DAVID version 6.8 [31, 32] to analyze their enriched GO (Gene Ontology) biological processes and KEGG (Kyoto Encyclopedia of Genes and Genomes) signaling pathways. p <0.05 and the number of enriched genes count ≥2 are used as the threshold for screening significant enrichment.

### 2.6 Target protein interaction analysis

Based on the obtained related target genes of AS-IV acting on CKD, the STRING database [33] was used to search for the interaction relationship between the corresponding proteins. Cytoscape Version 3.6.1 [34] was used to construct a visual network for the above-mentioned protein to protein interaction (PPI). The threshold for interaction screening is combined score = 0.4. Then the CytoNCA plug-in [35] was used to perform node degree analysis on the above-mentioned PPI network, and the parameters were set to without weight. Screen key proteins based on the ranking of node degrees in the network. At the same time, we also used the MCODE plug-in [36] to identify module genes in the network, with parameters set to default values and a threshold of score ≥5.

### 2.7 Network pharmacology and molecular docking analysis

Based on the components of AS-IV-target protein-pathway, cytoscape was used to construct a pharmacological network to demonstrate the pathway regulation mechanism of target proteins related to the treatment of CKD by AS-IV. In this study, the top 10 pathways (according to p value) in the pathway enrichment analysis were selected to construct the network.

The top 3 key proteins in the PPI network and the genes of the cAMP signaling pathway were selected as key targets. Download the structure file of the complex formed by the above target protein and other small molecules in the PDB database for subsequent research. Download the SDF format file of the molecular structure of AS-IV through the PubChem Compound database [37]. Convert it to PDB format using pymol for subsequent molecular docking analysis. Finally, Autodock (version 4.2.6) software [38] was used to perform molecular docking between key small molecules and targets.

### 2.8 NRK-52E proliferation detection

The CCK8 cell proliferation experiment was used to observe the proliferation of cells in each group after NRK-52E intervention with different UII concentrations for 24 hours. Group according to different UII concentrations: 0mol/L UII, 10^-10^mol/L UII, 10^-9^mol/L UII, 10^-8^mol/L UII, 10^-7^mol/L UII, 10^-6^mol/L UII. NRK-52E was cultured in complete culture medium (89% DMEM high-glucose medium, 10% fetal bovine serum, 1% penicillin-streptomycin double antibody), 37°C, 5% CO2 and 100% humidity cell culture incubator. Cells were passaged when confluence reached about 95%. When the cells reached the third passage and the confluence reached about 90%, the cells were planted in a 96-well plate and then cultured in a cell culture incubator for 24 hours. After observing that the cells are in an adherent state, 100 μL of UII of different concentrations were added to each well according to the experimental group and placed in a cell culture incubator for 24 hours. Add 10 μL of cell proliferation detection reagent to each well, and then place the 96-well plate in a cell culture incubator and incubate for 1–4 hours. After incubation, microplate reader was used to detect the OD value at 450nm in each well.

### 2.9 Determination of cAMP content by ELISA

The cell culture method is the same as above. Experimental groups were set as follow: control group, UII group (10^−8^ mol/L UII), UII + FSK group (10^−8^ mol/L UII+ Forskolin (cAMP agonist, 10 μmol/L)), UII + SB-611812 group (10^−8^ mol/L UII + SB-611812 (UIl receptor antagonist, 1 μmol/L)), UII + AS-IV group (10^−8^ mol/L UII + 40μmol/L AS-IV [39]), UII + H89 group (10^−8^ mol/L UII + H89 (cAMP inhibitor, 20 μmol/L)), UII + losartan group (10^−8^ mol/L UII + 10^-5^mol/L losartan). All cells were collected and lysed using RIPA lysis buffer after treatment. The lysed cells were centrifuged at 1000 r/min at 4°C for 20 min, the supernatant was collected, and the cAMP content was detected according to the instructions of the cAMP ELISA kit.

### 2.10 Cell immunofluorescence

After washing the slides with sterile PBS solution, fix the cells on the slides with 4% paraformaldehyde for 25 minutes at room temperature. The cells were then punched with 0.5% Triton for 10 minutes and blocked with 3% BSA for 30 minutes at room temperature. Place the cell sample on a slide on which primary antibodies (rabbit anti-GPR-14 antibody, rabbit anti-PKA antibody, rabbit anti-NF-κB antibody, rabbit anti-TGF-β antibody) have been dropped. Place the cell samples in a humidified box and incubate overnight in a 4°C refrigerator. The next day, the cell slides were washed with sterile PBS solution and placed in a darkroom for further processing. Place the cells onto a glass slide on which Alexa Fluor® 647 goat anti-rabbit IgG has been dropped, and incubate for 2 hours at room temperature in the dark. Add DAPI to counterstain the cells for 10 minutes and then add anti-fluorescence attenuation mounting medium dropwise to mount the slide. Place the cell sample under an oil lens and take pictures. Image-J was used to quantitatively calculate the fluorescence intensity of each indicator in each group.

### 2.11 Quantitative RT-PCR

Primers were designed in Pubmed and synthesized by Invitrogen Co., Ltd. The primer pair sequences (rat) are as follows:

**Table:** 

Gene Name		Gene Sequence (5’→3’)
GPR-14	Forward	GACTTCCTGACAATGCACGC
	Reverse	TTACGGTAACCCTTGGAGCG
NF-κB	Forward	GATCCTTTCGGAACTGGGCA
	Reverse	GGTATGGGCCATCTGCTGTT
TGF-β	Forward	ATGCCAACTTCTGTCTGGGG
	Reverse	CCCGGGTTGTGTTGGTTGTA
PKA	Forward	AAGAAAGGCAGCGAAGTGGAG
	Reverse	ATTACTCGGGGGAGGGTTCT
Actb	Forward	TGTCACCAACTGGGACGATA
	Reverse	GGGGTGTTGAAGGTCTCAAA

A total RNA extraction kit was used to extract RNA from each group, and the total RNA concentration was measured. Reverse transcription was performed using a reverse transcription kit to synthesize cDNA. Using cDNA as a template, use a qPCR amplification kit to amplify the cDNA, and quantitatively detect the fluorescence of the amplified product. The results are calculated using relative quantification 2-ΔΔCT to obtain the expression level of the target gene mRNA.

### 2.12 Western blotting

The protein of cells was collected, and the protein content was determined by BCA protein quantification method. Proteins were separated by 8% SDS-PAGE electrophoresis. Use a Bio-Rad electroporation instrument to transfer the proteins in the gel to a PVDF membrane. Then, wash the membrane three times with TBS-T for 5 minutes each time. The above PVDF membrane was blocked with 5% skimmed milk powder at room temperature for 1 hour. The membrane was incubated with primary antibodies (rabbit anti-GPR-14 antibody, rabbit anti-PKA antibody, rabbit anti-NF-κB antibody, rabbit anti-TGF-β antibody) overnight at 4°C. The next day, wash the membrane 3 times with TBS-T, 5 minutes each time. The membrane was incubated with horseradish peroxidase-labeled secondary antibody for 1 hour at room temperature. After washing the membrane with TBS-T, perform subsequent processing in a darkroom. Mix the ECL chem-iluminescence reagent and cover it on the membrane. The film was exposed in a dark box. Use Alpha-view software to scan and analyze the results.

### 2.13 Data analysis and statistics

Statistical analysis was performed using GraphPad Prism 9.5.1 software and IBM SPSS Statistics26. Measurement data are expressed in mean ± SD. Comparisons between multiple groups were performed using one-way analysis of variance. Pairwise comparisons between groups were performed using the LSD-t test. The difference was considered statistically significant when P<0.05.

## 3 Results

### 3.1 Prediction results of AS-IV target protein

By using BATMAN, SwissTargetPrediction, and ETCM databases for prediction, 58, 44, and 4 target proteins for AS-IV to exert pharmacological effects were predicted, respectively. Finally, a total of 101 target proteins were obtained by combining the above results (S1 Fig).

### 3.2 Screening of CKD-related genes

**Fig 1:**
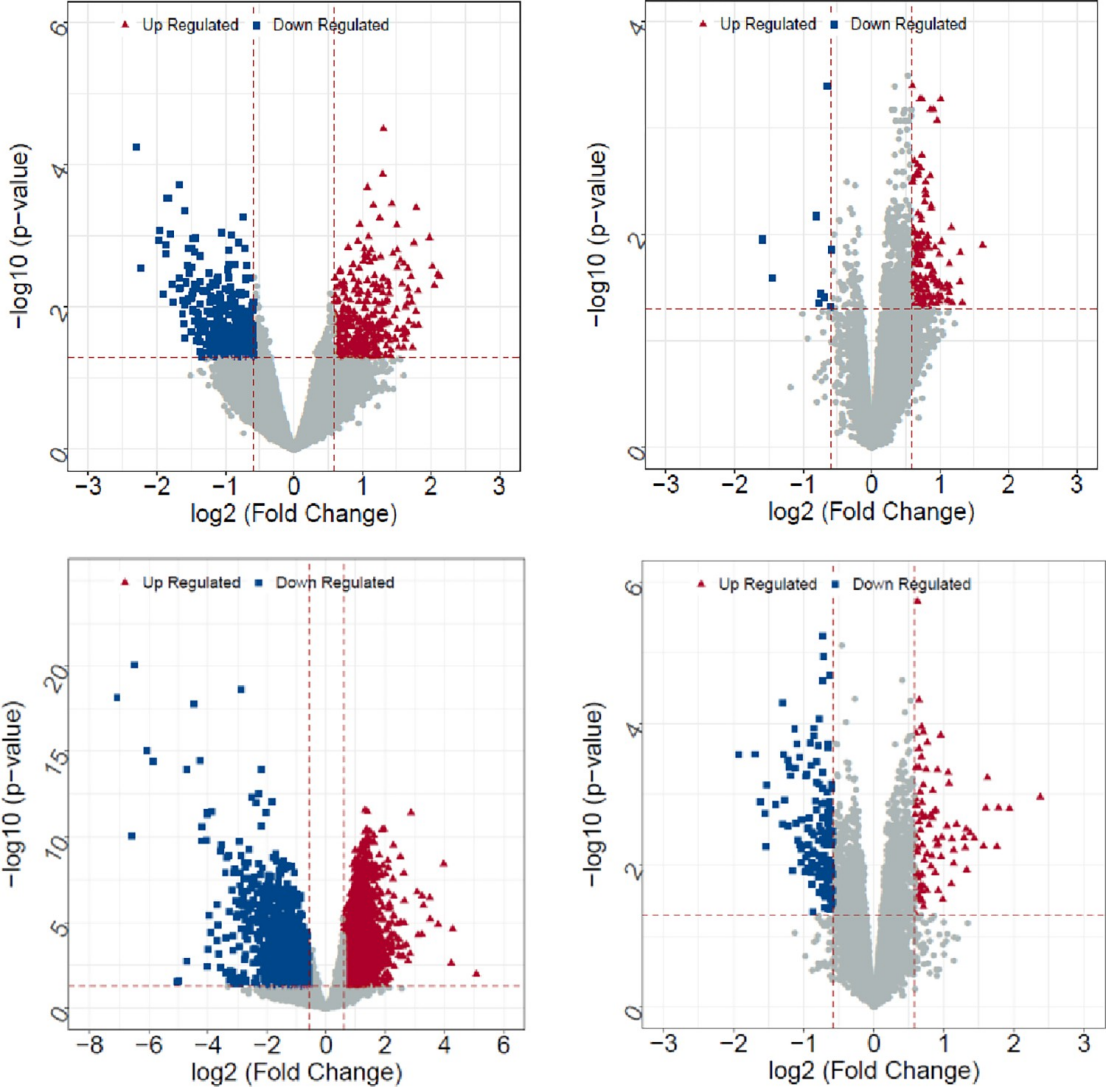
Volcano plot of differentially expressed genes. Upper left, GSE15072; upper right, GSE62792; lower left, GSE66494; lower right, GSE98603.

After screening four data sets (GSE15072, GSE62792, GSE66494, and GSE98603), 685 (319 up-regulated, 366 down-regulated), 157 (148 up-regulated, 9 down-regulated), 8403 (6730 up-regulated, 1673 down-regulated), and 237 (93 up-regulated, 144 down-regulated) DEGs were obtained respectively (Fig 1).

### 3.3 Function and pathway enrichment analysis

The 101 target proteins of AS-IV obtained above and the DEGs screened from the four data sets (GSE15072, GSE62792, GSE66494, GSE98603) were intersected, and 7, 0, 49, and 1 DEGs were obtained respectively. The results obtained from these several data sets were then combined to obtain 54 target genes (S2 Fig)

**Fig 2:**
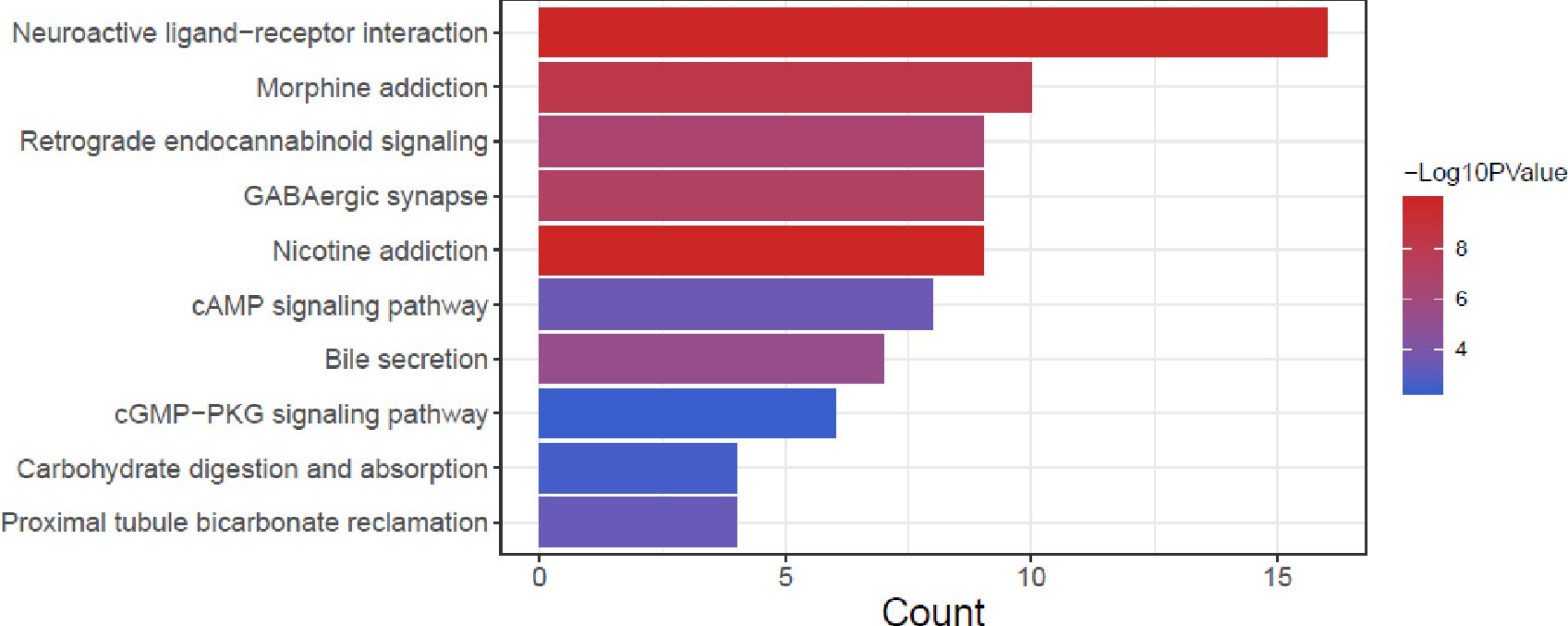
KEGG pathway of AS-IV acting on 54 disease-related genes. The length of the bar in the figure indicates the number of enriched genes, and the color from blue to red indicates the significance. The smaller the p.value, the more significant.

Functional and pathway enrichment analysis was performed on the 54 target genes obtained above, and a total of 105 significantly enriched GO biological functions and 21 KEGG signaling pathways were obtained. The biological functions enriched by GO analysis mainly include ion transmembrane transport, γ-aminobutyric acid signaling pathway, positive regulation of heterotypic intercellular adhesion, positive regulation of ERK1 and ERK2 cascades, etc (S3 Fig). Significantly enriched KEGG pathways include neuroactive ligand-receptor interaction, cAMP signaling pathway, proximal tubule bicarbonate recycling, cGMP-PKG signaling pathway, etc. (Fig 2). According to *p* value, we presents the top 10 functions and pathways (S1 Table).

### 3.4 Protein interaction analysis

In this study, a total of 47 interacting proteins were screened (S2 Table), including 129 relationship pairs (see S3 Table for details of the connectivity ranking). The top three connected proteins are ALB, VEGFA, and AKT1. Then the PPI network is identified and two sub-modules are obtained based on threshold screening.

### 3.5 Pharmacological network construction and molecular docking

**Fig 3:**
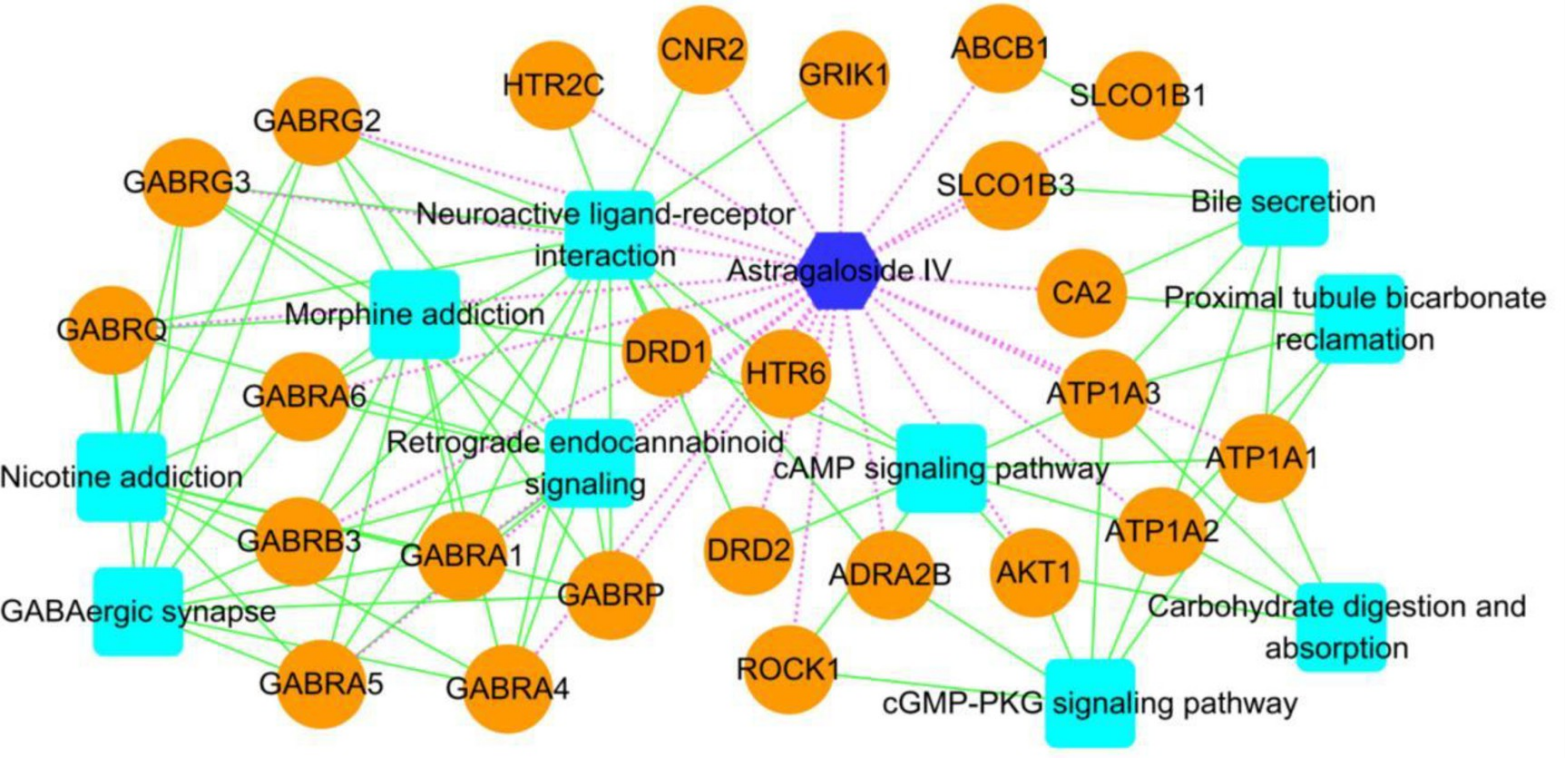
Network diagram of network pharmacology analysis. The blue hexagon is the component of AS-IV. The orange circle represents the target gene, the light blue square represents the signaling pathway; the green line represents the gene participating in a certain pathway, and the light purple dotted line represents the targeting effect of the component and the target gene protein.

The constructed AS-IV component-target protein-pathway network diagram contains 37 nodes and 82 relationships (Fig 3). These include 1 chemical component node, 25 target protein nodes and 10 pathway nodes (S4 Table).

**Fig 4:**
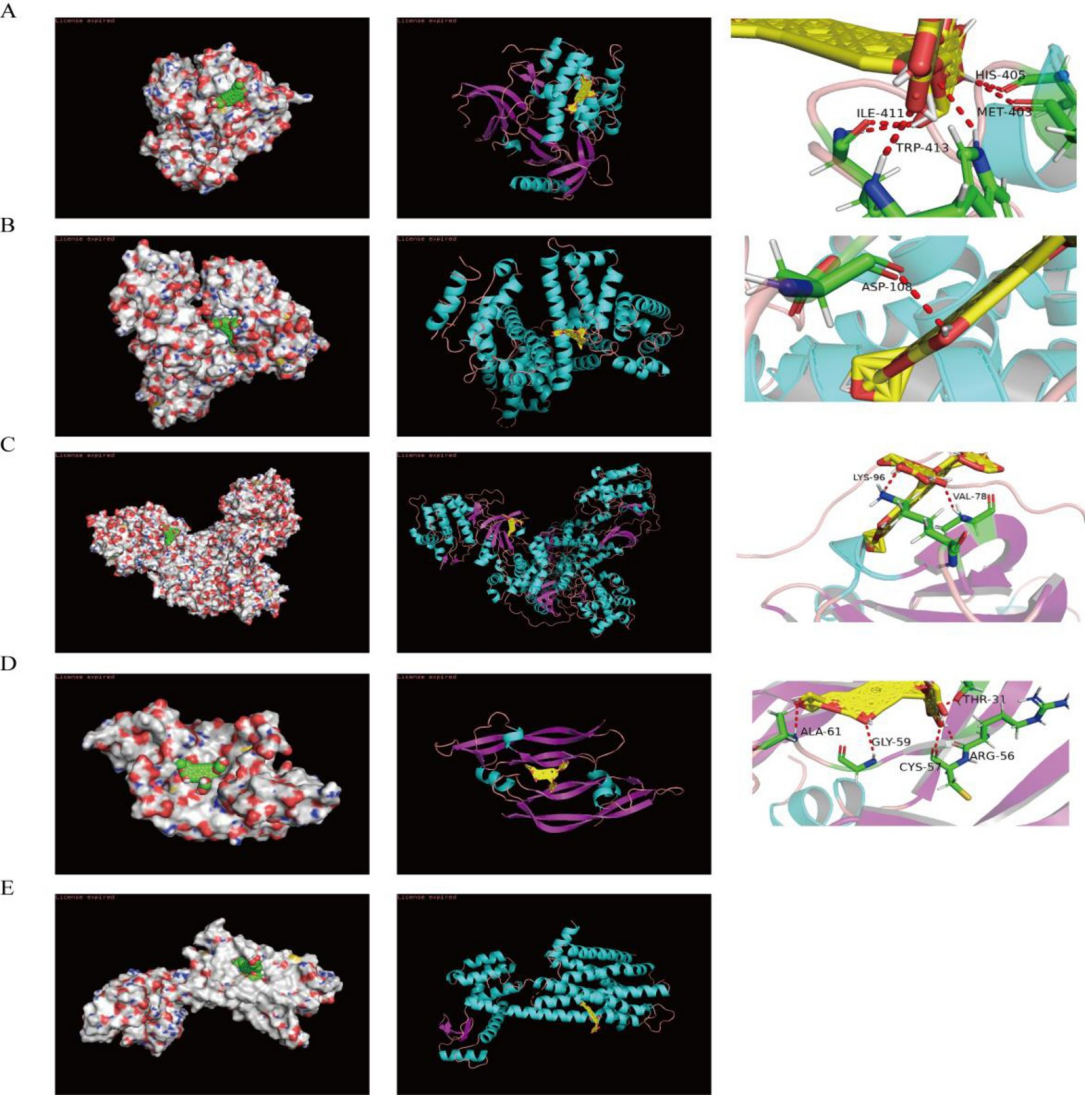
Schematic diagram of molecular docking. A, B, C, D, and E represent the molecular docking results of AKT1, ALB, ROCK1, VEGFA, and DRD2, respectively. The left column shows the global diagram of simulated docking, in which the green sphere represents the ligand small molecule AS-IV; The middle column indicates the results for each receptor, in which yellow indicates the ligand small molecule AS-IV; The column on the right is a partial three-dimensional diagram of simulated docking, in which yellow represents the ligand small molecule AS-IV, and green represents the bound protein amino acid residues. The surrounding text indicates amino acid residues, and the red dashed lines indicate interacting hydrogen bonds.

Molecular docking analysis was conducted using the top 3 key proteins in the PPI network (ALB, VEGFA, AKT1) and cAMP signaling pathway proteins (ROCK1, DRD2), as well as AS-IV. Download the crystal structure of the complex of ALB, VEGFA, AKT1, ROCK1, DRD2 and other ligands from the PDB database. Their PDB IDs are 6YG9, 1MKK, 3O96, 3V8S, and 6CM4, respectively. Water molecules and small molecules were removed from these structures and the protein 3D structure was isolated. Then, based on the principle that the smaller the Binding Energy, the higher the possibility of binding, the model with the smallest Binding Energy was selected as the best model and a visual simulation was performed (Fig 4). Among them, the minimum binding energy of AS-IV with ALB, VEGFA, AKT1, ROCK1, and DRD2 are: -8.91, -12.87, -9.24, -5.68, and -8.77 respectively.

### 3.6 Effects of different concentrations of UII on NRK-52E proliferation

**Fig 5:**
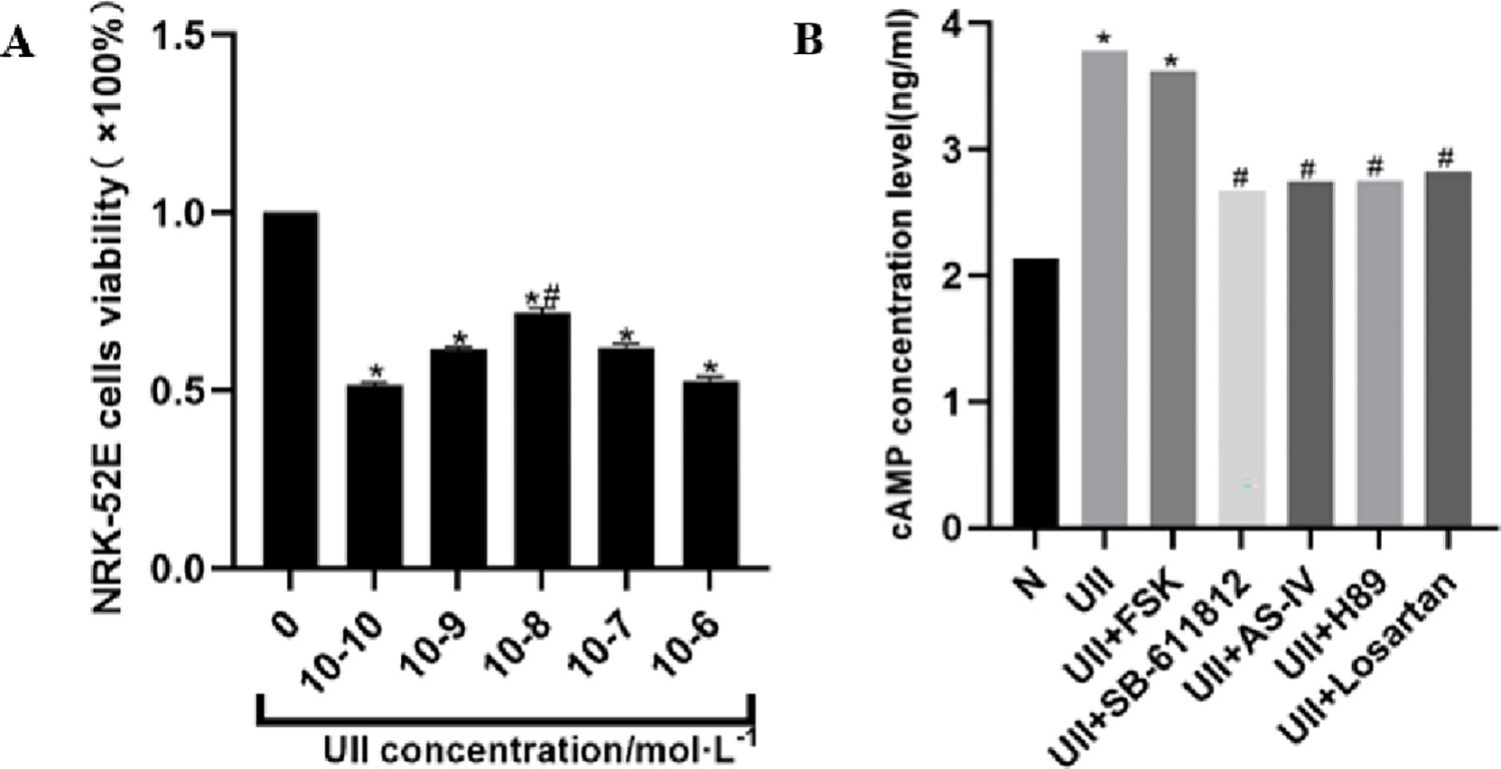
Effects of AS-IV on cAMP content in NRK-52E. A, Viability of NRK-52E cells treated with different concentrations of U II. *P<0.05, compared with control group; #P<0.05, compared with 10^−10^ mol/L, 10^−9^ mol/L, 10^−7^ mol/L and 10^−6^ mol/L UⅡ group, respectively, n ≥3. B, The changes in intracellular cAMP concentration after NRK-52E cells are treated with UII and UII in combination with various signal transduction pathway regulators. *P<0.05, compared with the N group, #P<0.05, compared with the UII group, n ≥3.

The results showed that there was no linear correlation between UⅡ concentration and cell viability. Compared with the control group (0 mol/L UII), the cell viability of the 10^-10^mol/L, 10^-9^mol/L, 10^-8^mol/L, 10^-7^mol/L, and 10^-6^mol/L UⅡ groups all decreased (P<0.05, Fig 5A). It indicated that UII treatment will inhibit cell viability of NRK-52E. And the treatment of 10^−8^ mol/L UII showed less effect on cell viability, which indicates 10^−8^ mol/L of UII is a relative safe concentration for UII-mediated renal tubular epithelial cell injury study. Therefore, 10^−8^ mol/L of UII was used in subsequent experiments.

### 3.7 Effects of AS-IV on cAMP content in NRK-52E

Compared with the control group, the cAMP levels in NRK-52E cells in the UII group and the UII + FSK group were significantly increased (P<0.05). However, compared with the UII group, the cAMP levels in the UII + SB-611812 group, UII + AS-IV group, UII + H89 group and UII + losartan group were decreased significantly (P<0.05). The results showed that under the treatment of SB-611812, AS-IV, H89 and losartan, the upregulation of cAMP in NRK-52E cells by UII can be inhibited (Fig 5B).

### 3.8 Cellular immunofluorescence detection of the impact of AS-IV on key genes

**Fig 6:**
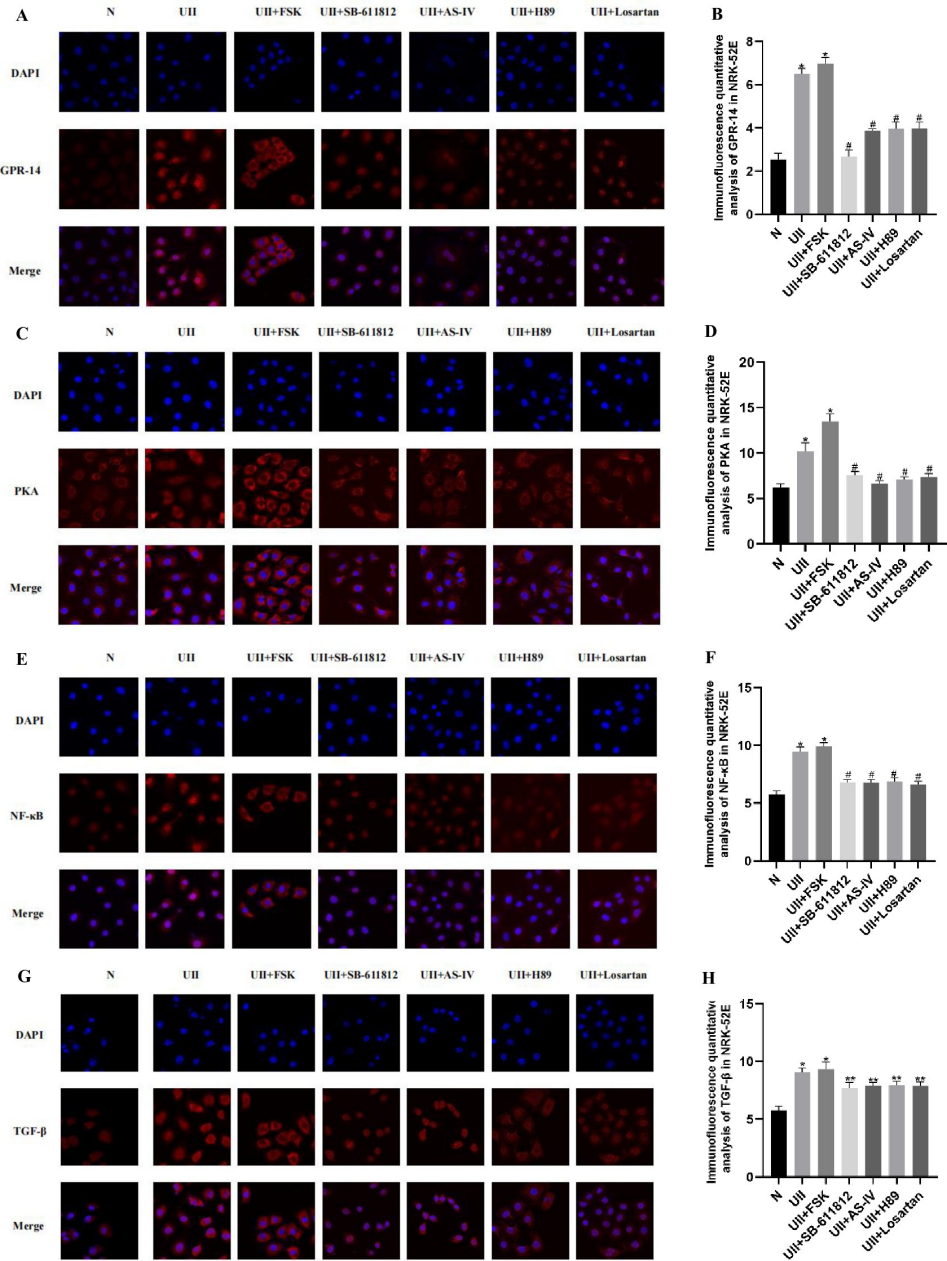
The expression of key genes after AS-IV treatment, detected by immunofluorescence. A, C, E, G, Laser confocal images of NRK-52E cells in each group (10×63 times, eyepiece 10 times, oil lens 63 times. B, D, F, H, Quantitative immunofluorescence analysis of NRK-52E cells in each group. *P<0.05 compared with N group, ^#^P<0.05 compared with UII group, n ≥3.

The results showed that GPR-14 (Fig 6A and 6B), PKA (Fig 6C and 6D), NF-κB (Fig 6E and 6F), and TGF-β (Fig 6G and 6H) were expressed in scattered spots in the cytoplasm and nucleus of NRK-52E. Relatively lower GPR-14, PKA, NF-κB, and TGF-β expression were found in NRK-52E in the control group. After treatment with UII, intracellular GPR-14, PKA, NF-κB, and TGF-β were significantly increased (P<0.05). After cells were treated with UII + SB-611812, UII + AS-IV, UII + H89 and UII + losartan, respectively, compared with the UII group, the expressions of GPR-14, PKA, NF-κB and TGF-β were significantly decreased (P<0.05). Combined with the ELISA results, it is suggested that the cAMP/PKA signaling pathway may be involved in UII-induced renal tubular epithelial cell injury. AS-IV and losartan may downregulate the cAMP/PKA signaling pathway to protect renal tubular epithelial cells.

### 3.9 Quantitative RT-PCR to verify the impact of AS-IV on key genes

**Fig 7:**
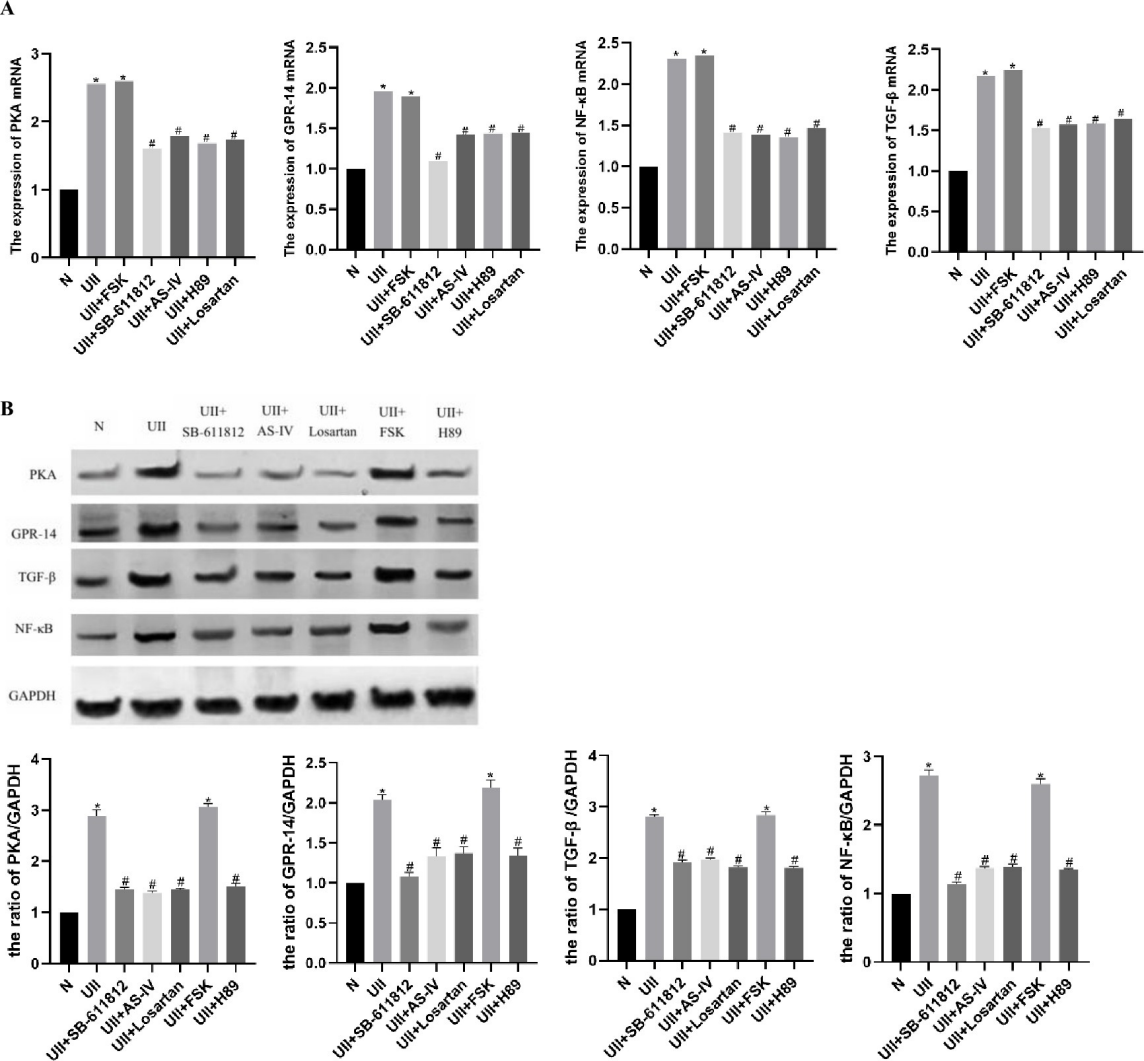
The expression of key genes after AS-IV treatment, detected by Q-PCR and Western blot. A, The mRNA expression levels of PKA, GPR-14, NF-κB, and TGF-β in each group after NRK-52E intervention. B, The protein expression of PKA, GPR-14, NF-κB, and TGF-β in each group after NRK-52E intervention. *P < 0.05 compared with the N group, ^#^P<0.05, compared with the UII group.

Experimental results showed that NRK-52E in the control group had relatively lower GPR-14, PKA, NF-κB, and TGF-β mRNA expression. After stimulation with UII, the expression levels of intracellular GPR-14, PKA, NF-κB, and TGF-β mRNA were significantly increased (P<0.05). After treatment with UII + SB-611812, UII + AS-IV, UII + H89 and UII + losartan, respectively, compared with the UII group, the expression of GPR-14, PKA, NF-κB, TGF-β mRNA were decreased significantly (P<0.05). Combined with the ELISA results, it is suggested that the cAMP/PKA signaling pathway may be involved in UII-induced renal tubular epithelial cell injury. AS-IV and losartan may downregulate the cAMP/PKA signaling pathway to protect renal tubular epithelial cells (Fig 7A).

### 3.10 Western blot to verify the impact of AS-IV on key genes

The results showed that the control group NRK-52E had relatively lower GPR-14, PKA, NF-κB, and TGF-β protein expression. After treatment with UII, the expression levels of intracellular GPR-14, PKA, NF-κB, and TGF-β proteins were significantly increased (P<0.05). After cells were treated with UII + SB-611812, UII + AS-IV, UII + H89 and UII + losartan, respectively, compared with the UII group, the protein expression levels of GPR-14, PKA, NF -κB and TGF-β were significantly decreased (P<0.05). Combined with the ELISA results, it is suggested that the cAMP/PKA signaling pathway may be involved in UII-induced renal tubular epithelial cell injury. AS-IV and losartan may downregulate the cAMP/PKA signaling pathway to protect renal tubular epithelial cells (Fig 7B).

## 4 Discussion

The prevalence of CKD among adults worldwide is as high as 10–15% [40]. And its incidence rate is increasing year by year in both developed and developing countries [41], but there is still a lack of effective intervention methods. Our previous research found that AS-IV has a certain protective effect on renal tubular epithelial cell damage in diabetic nephropathy [42].

This study used network pharmacology research methods to explore the molecular mechanism of AS-IV in delaying the progression of kidney disease. Through differential analysis, we screened a total of 54 genes related to AS-IV treatment of CKD. These genes are enriched in 21 pathways, including cAMP signaling pathway, neuroactive ligand-receptor interaction, cGMP-PKG signaling pathway, and so on. Among them, the cAMP signaling pathway is the key pathway for AS-IV to treat CKD. Subsequently, we conducted molecular docking analysis of the top three key proteins in the PPI network (ALB, VEGFA, AKT1) and key proteins in cAMP pathway (ROCK1, DRD2) with AS-IV, respectively. We found that AS-IV molecules can be well embedded in the active pockets of the above five key gene proteins (ALB, VEGFA, AKT1, ROCK1, and DRD2).

Studies have shown [43] that the cAMP/PKA signaling pathway is involved in regulating the process of tissue and organ fibrosis. Activating the cAMP/PKA signaling pathway can induce the expression of downstream TGF-β and other factors, thereby promoting the progression of renal fibrosis [44]. The cAMP/PKA signaling pathway can regulate nuclear transcription factor-κB (NF-κB) and activate the NLRP3 inflammasome at the transcriptional level. Studies have shown that Intermedin (IMD, a small peptide belonging to the calcitonin/calcitonin-generating peptide family) can inhibit the production of reactive oxygen species (ROS) by down-regulating the cAMP-PKA signaling pathway, thereby reducing renal fibrosis caused by unilateral ureteral obstruction (UUO) [45]. Renal fibrosis is primarily driven by inflammatory cytokines, including transforming growth factor-β (TGF-β) [46, 47]. At the same time, NF-κB is a major regulator of many pro-inflammatory and pro-fibrotic molecules [48]. Therefore, we used TGF-β and NF-κB as indicators of cellular fibrosis. The results of this study show that UII can induce the proliferation of renal tubular epithelial cells by upregulating the cAMP/PKA signaling pathway, leading to an increase in the inflammatory injury factors NF-κB and TGF-β, and promoting renal tubular epithelial cell damage. It is suggested that the cAMP/PKA signaling pathway plays an important role in the progression of kidney disease.

As a new vasoactive peptide, UII plays an important role in a variety of pathophysiological processes. Studies have proven [14, 49] that the combination of UII and GPR14 can not only regulate vascular tone, but also promote cell proliferation, epithelial cell-to-mesenchymal transition, and extracellular matrix accumulation in the kidney. And it also promotes extracellular calcium influx in the renal tubular epithelium and the release of calcium ions in the sarcoplasmic reticulum, which in turn leads to an increase in intracellular calcium and activates calcineurin. Eventually, the transcription factor NFAT3 is dephosphorylated and transferred into the nucleus, regulating gene expression and producing a pro-proliferative effect. In addition, UII can also promote the proliferation of renal interstitial fibroblasts in a dose-dependent manner and affect the cell cycle. And promotes renal fibrosis by regulating inflammatory signaling pathways such as JAK2/STAT3. Recent studies have shown that UII can induce pyroptosis by regulating the NLRP3-Caspase1 pathway [50]. And it can enhance the expression and secretion of TGF-β in renal tubular epithelial cells, and induce the up-regulation of alpha-smooth muscle actin (α-SMA), fibroblast specific protein 1 (FSP1), fibronectin (FN) and collagen IV (Col Ⅳ) in human renal tubular epithelial cells, thereby promoting the occurrence and development of fibrosis [11–13]. Combined with the results of this study, it is suggested that UII and TGF-β have similar pathological effects. UII can induce the expression of NF-κB and TGF-β in NRK-52E cells by upregulating the cAMP/PKA signaling pathway, thereby inducing inflammatory damage of renal tubular epithelial cells and promoting the occurrence and development of fibrosis.

Studies have found that by down-regulating the AKT/GSK-3β signaling pathway, AS-IV can reduce the expression of α-SMA, type I collagen and fibronectin induced by TGF-β1 and inhibits the epithelial-to-mesenchymal transition of rat renal tubular epithelial cells and delays renal fibrosis [51]. At the same time, AS-IV can reduce the urinary protein excretion rate of rat models of diabetic nephropathy and inhibit the proliferation of glomerular mesangial cells in rats, thereby delaying renal fibrosis. In the UUO model of rats with obstructive nephropathy, AS-IV can inhibit the infiltration of inflammatory cells and improve the survival rate of renal tubular cells by regulating the TLR4/NF-кB, TGF-β/Smad3, and Wnt/β-catenin signaling pathways and prevent cell apoptosis, thereby delaying the progression of renal fibrosis [19, 52–54]. Our preliminary research has found that AS-IV has a certain role in inhibiting inflammatory mediators and improving renal fibrosis in a rat model of diabetic nephropathy. In this study, it was found that AS-IV can down-regulate the cAMP/PKA signaling pathway and inhibit the expression of inflammatory factors in renal tubular epithelial cells, thus exerting its renal protective effect. This result provides a more powerful experimental basis for exploring the treatment of AS-IV in kidney disease, and provides certain evidence for the clinical promotion of AS-IV.

This study preliminarily analyzed the mechanism of AS-IV in improving renal fibrosis based on network pharmacology methods and molecular docking technology, and selected key pathways for in vitro experimental verification. The results show that AS-IV can exert a renal protective effect through different signaling pathways, reflecting the characteristics of the combined effects of AS-IV on multiple pathways. This study provides a scientific basis for the clinical application of AS-IV in the treatment of renal fibrosis, and also provides a new direction for exploring the potential mechanism of action of AS-IV. The experimental verification of this study found that UII can induce damage to renal tubular epithelial cells to a certain extent, and AS-IV can improve this damage. However, the relationship between other discovered signaling pathways and other downstream target genes such as ROCK1, DRD2, etc. still needs further experimental verification. In the future, we will continue to conduct experimental verification of target genes downstream of this pathway. In summary, through this network pharmacology analysis, we have obtained more new targets for AS-IV in the treatment of renal fibrosis, and also demonstrated that network pharmacology is one of the powerful tools for exploring the mechanism of drug action.

## 5. Conclusions

AS-IV may improve UII-mediated renal tubular epithelial cell damage by down-regulating the cAMP/PKA signaling pathway. Further animal experiments will need to validate the results and conclusions obtained from network pharmacology and cell biology, with the aim of corroborating these findings at the animal level. This may provide a new direction for the study and treatment of renal tubular epithelial cell damage.

## Supporting information

S1 Raw images (PDF)

S1 Table The enrichment results of 54 target genes. (XLSX)

S2 Table PPI network relationships. (XLSX)

S3 Table PPI network protein connectivity. (XLSX)

S4 Table Astragaloside-target protein-pathway network. (XLSX)

S1 Fig Target prediction results of astragaloside IV. Red diamond, predicted tool or database; green circle, target gene name.
(PDF)

S2 Fig Venn diagram of the intersection of astragaloside IV target protein and differential genes in each data set. (PDF)

S3 Fig The astragaloside IV component acts on GO BPs that are significantly enriched in 54 disease-related genes. Red dots, enriched up-regulated genes; Blue origin, enriched down-regulated genes; The middle strip, the pathway z-score, reflects the up- and down-regulation of the pathway to a certain extent; Red strip, z-score is greater than 0, indicating that the pathway is activated; Blue strip, z-score less than 0, indicates that the pathway is inhibited.
(PDF)
