# Supplementary figures and images for: Integrating network pharmacology and experimental validation to explore the pharmacological mechanism of Astragaloside IV in alleviating urotensin II-mediated renal tubular epithelial cell injury
Source: PLoS One. 2024 Dec 20;19(12):e0310210. doi: 10.1371/journal.pone.0310210 (PMC11661590; doi:10.1371/journal.pone.0310210)

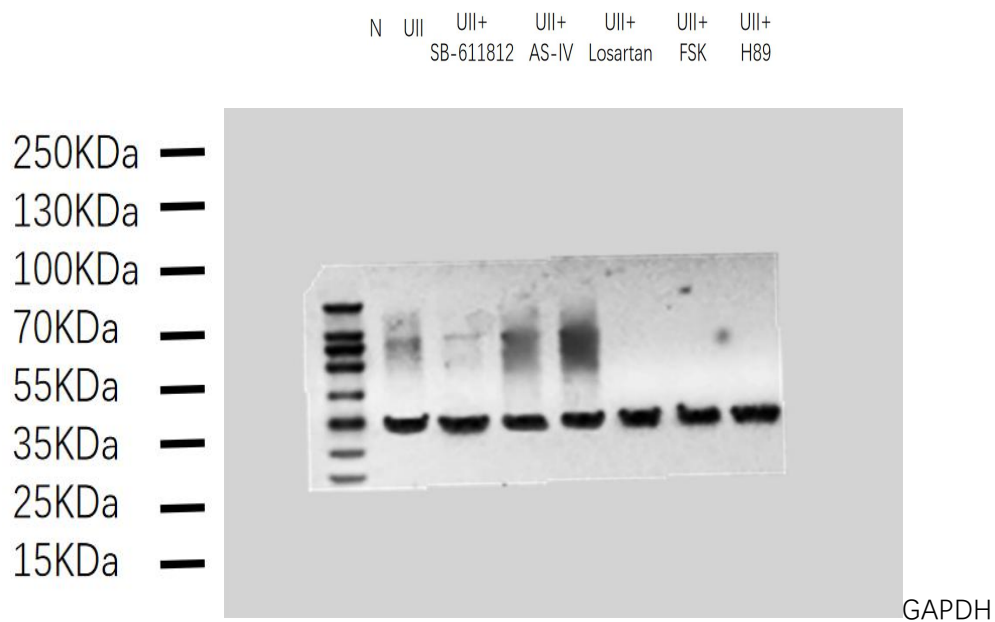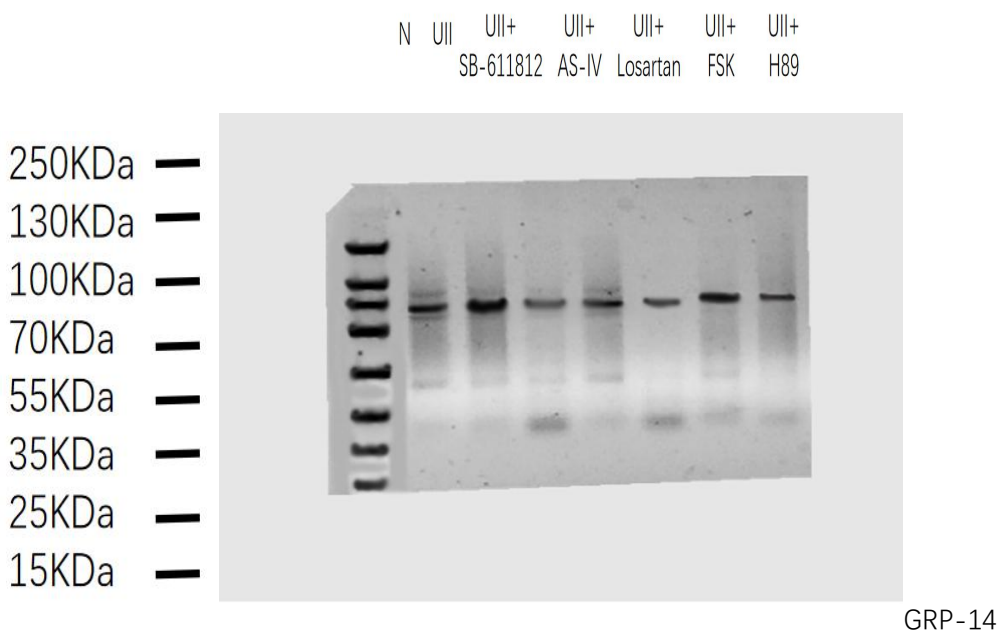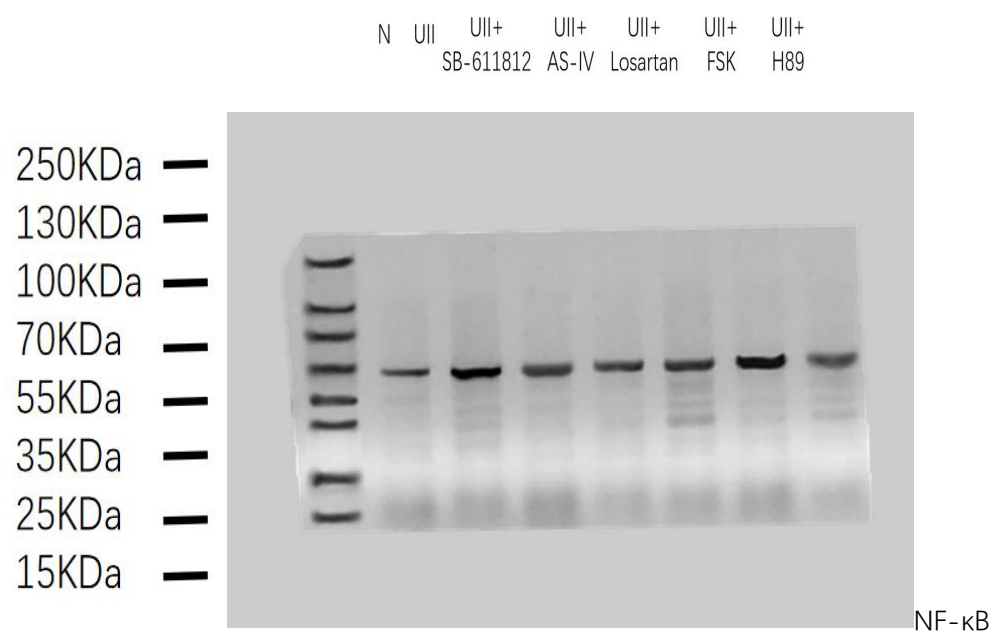

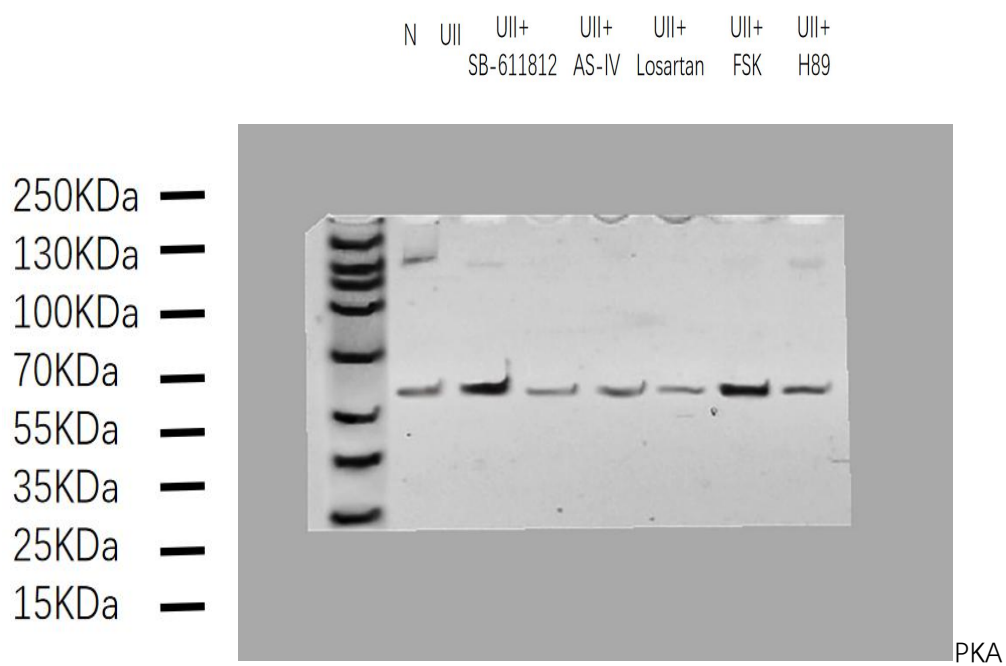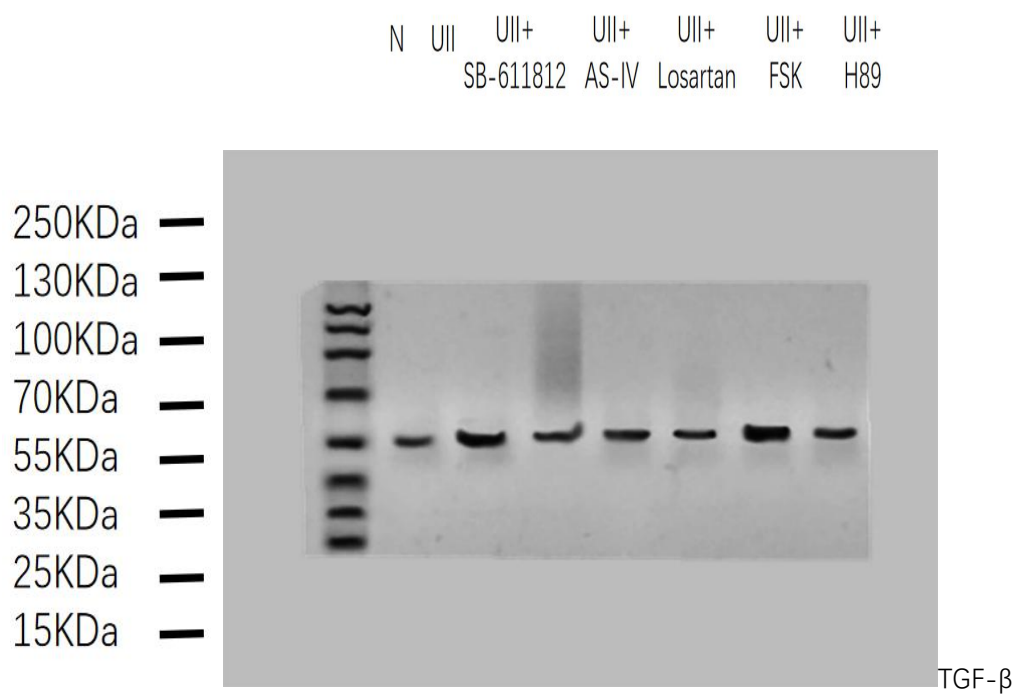

Supplement: S1 Raw images — (PDF) [file pone.0310210.s001.pdf]

### Supplementary Figure 1

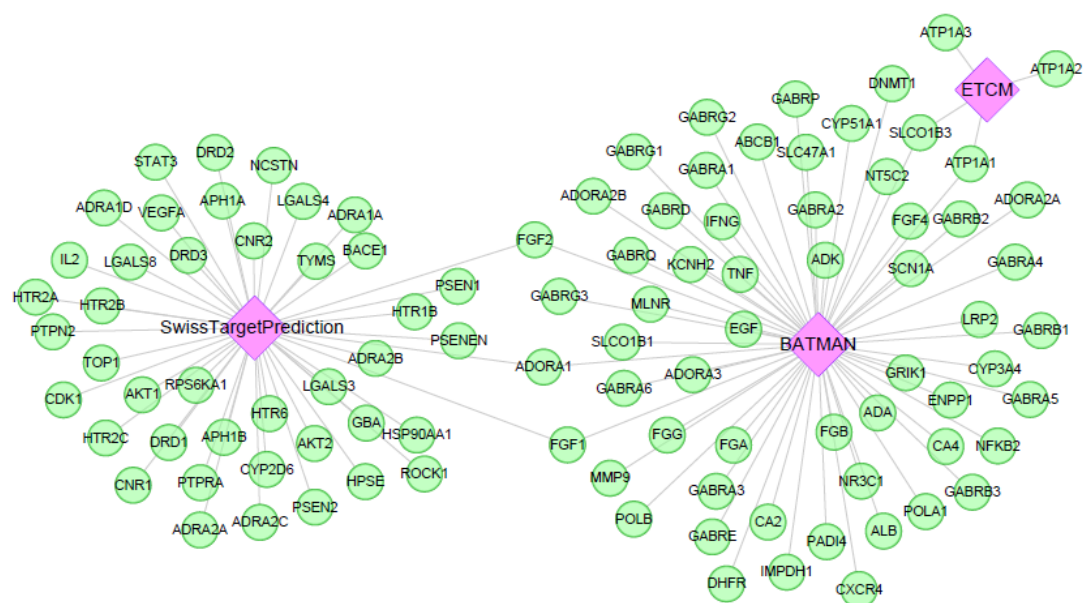

Supplement: S1 Fig — Red diamond, predicted tool or database; green circle, target gene name. (PDF) [file pone.0310210.s006.pdf]

## Supplementary Figure 2

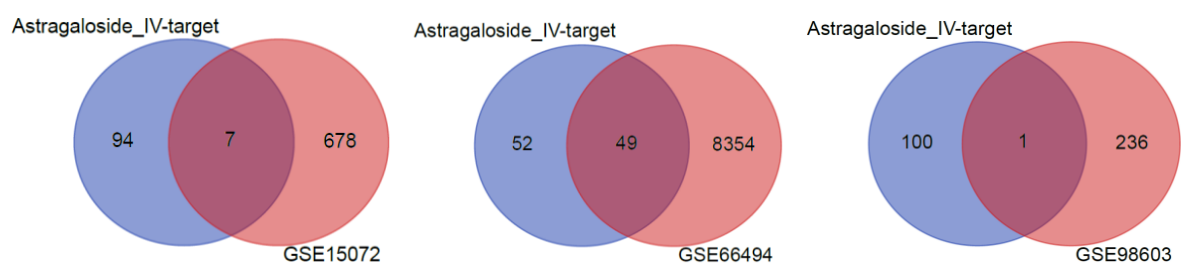

Supplement: S2 Fig — (PDF) [file pone.0310210.s007.pdf]
