# Supplementary material for: Integrating network pharmacology and experimental validation to explore the pharmacological mechanism of Astragaloside IV in alleviating urotensin II-mediated renal tubular epithelial cell injury
Source: PLoS One. 2024 Dec 20;19(12):e0310210. doi: 10.1371/journal.pone.0310210 (PMC11661590; doi:10.1371/journal.pone.0310210)
Supplement: S3 Fig — Red dots, enriched up-regulated genes; Blue origin, enriched down-regulated genes; The middle strip, the pathway z-score, reflects the up- and down-regulation of the pathway to a certain extent; Red strip, z-score is greater than 0, indicating that the pathway is activated; Blue strip, z-score less than 0, indicates that the pathway is inhibited. (PDF) [file pone.0310210.s008.pdf]

Supplementary Figure 3

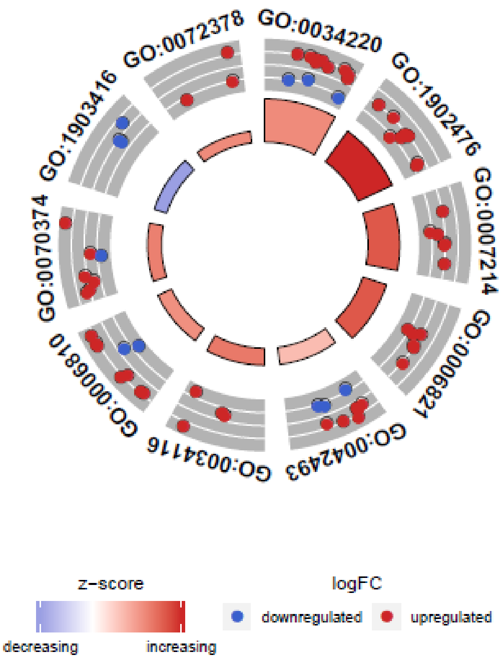

ID	Description
GO:0034220	ion transmembrane transport
GO:1902476	chloride transmembrane transport
GO:0007214	gamma−aminobutyric acid signaling pathway
GO:0006821	chloride transport
GO:0042493	response to drug
GO:0034116	positive regulation of heterotypic cell−cell adhesion
GO:0006810	transport
GO:0070374	positive regulation of ERK1 and ERK2 cascade
GO:1903416	response to glycoside
GO:0072378	blood coagulation, fibrin clot formation
